# A case report of radiotherapy for head and neck cancer in a pregnant woman: dosimetric optimization and *in vivo* dosimetry

**DOI:** 10.1016/j.tipsro.2026.100387

**Published:** 2026-02-17

**Authors:** Axel Martin, David Thibouw, Laurent Delcoudert, Léone Aubignac, Karine Peignaux, Christelle Huet, Magali Edouard, Jérémy Baude, Igor Bessieres

**Affiliations:** a Université Bourgogne Europe, Centre Georges-François Leclerc, Unicancer, Département de Physique Médicale, 21000 Dijon, France; b Université Bourgogne Europe, Centre Georges-François Leclerc, Unicancer, Département de Radiothérapie, 21000 Dijon, France; c French Nuclear Safety and Radiation Protection Authority (ASNR), France

**Keywords:** Pregnant woman, Head and neck cancer, Dosimetric optimization, *Invivo* dosimetry

## Abstract

**Background:**

Radiotherapy during pregnancy is a rare and ethically complex requiring careful management of fetal exposure. Although peripheral doses from head and neck radiotherapy are typically low, fetal irradiation carries a potential developmental or carcinogenic risk. Accurate dose estimation and optimization are essential when radiotherapy cannot be postponed.

**Case presentation:**

A 27-year-old woman, 26 weeks pregnant, required adjuvant radiotherapy for a left parotid adenoid cystic carcinoma (pT4a N3b, R1). To minimize fetal exposure, a one-month pre-treatment period was dedicated to dosimetric optimization. Three clinically equivalent VMAT plans were compared using different collimator angles (15°/345°, 80°/30°, and 80°/100°). Out-of-field dose measurements were assessed using a calibrated ionization chamber positioned in a slab phantom at varying depths and distances simulating fetal position and progression of pregnancy. All plan showed low out-of-field dose, below 100 mGy. The plan with 80°/100° collimator angles (Popt2) consistently showed the lowest out-of-field dose and was therefore selected for treatment. A lead apron was added during treatment. *In vivo* dosimetry was performed using radiophotoluminescent (RPL) detectors placed weekly under the lead apron at the uterine fundus projection. Over 10 monitored fractions, single-session doses averaged 1.25 mGy, and cumulative five-session doses reached 5.9–6.7 mGy. Total extrapolated fetal dose was approximately 41.0 mGy, closely matching the phantom-based estimate of 31.1 mGy.

**Conclusions:**

This unprecedented case in our institution demonstrates the feasibility of safely delivering radiotherapy during pregnancy through multidisciplinary coordination and meticulous dose management. Phantom-based optimization and *in vivo* dosimetry confirmed that the fetal dose remained below the 100 mGy threshold set by international guidelines for deterministic effects. The patient completed treatment without complications and delivered a healthy infant at term. This experience provided the foundation for a standardized dosimetric approach within our institution for future cases involving pregnancy and radiotherapy.

**Background:**

Radiotherapy during pregnancy is rare and presents significant ethical and technical challenges dure to fetal radiation exposure. The nature of these effects depends on both the dose level and the stage of pregnancy [1]. Although postponing treatment until after delivery is preferable whenever possible, radiotherapy can be administered during pregnancy under certain conditions. The International Commission on Radiological Protection (ICRP) has established that, for an absorbed dose below 100  mGy, no risk to fetal development is expected [2] and medical termination of pregnancy is not justified regarding radiation risks. Nevertheless, fetal dose optimization remains essential, as stochastic risks may exist even at low exposure levels.

## Highlights

• Fetal dose optimization and measurement for head and neck RT during pregnancy.
• VMAT collimator angle adjustment proved effective for fetal dose reduction.
• Phantom and *in vivo* dosimetry showed consistent fetal dose estimates.
• Lead apron shielding provided additional 5–13% fetal dose reduction.
• Established reproducible workflow for fetal dose estimation in RT.

## Background

Radiotherapy during pregnancy is rare and presents significant ethical and technical challenges dure to fetal radiation exposure. The nature of these effects depends on both the dose level and the stage of pregnancy [1]. Although postponing treatment until after delivery is preferable whenever possible, radiotherapy can be administered during pregnancy under certain conditions. The International Commission on Radiological Protection (ICRP) has established that, for an absorbed dose below 100  mGy, no risk to fetal development is expected [2] and medical termination of pregnancy is not justified regarding radiation risks. Nevertheless, fetal dose optimization remains essential, as stochastic risks may exist even at low exposure levels.

The most frequently reported treatment cases involve tumors located outside the abdominal region, notably in the parotid gland [3], brain [4], head and neck [5], or breast [6], including conformal or intensity modulated radiotherapy, with systematic estimation of fetal dose down to the ICRP 100  mGy limit. In such scenarios, the fetal dose is considered an out-of-field or peripheral dose, which is relatively low compared to the therapeutic dose. This dose is attributable to three main sources: scatter within the patient, scatter within the linear accelerator, and direct leakage from the accelerator head [7]. The relative contribution of each component varies depending on several factors, such as the distance between the fetus and the isocenter, the patient’s body habitus, treatment beam configuration, and the specific model of the linear accelerator [8]. Proton therapy is also an interesting alternative, particularly because of its superiority in sparing organs at risk (OAR). Some feedback from clinical experience has shown a significant reduction in fetal dose while maintaining equivalent or even superior treatment quality. Nevertheless, the main limitation of this technique lies in its accessibility [9].

Accurate estimation and minimization of fetal dose are critical and complex, as treatment planning systems do not reliably calculate out-of-field exposure [10], [11], [12].

This case represents our institution’s first experience managing a pregnant patient external beam radiotherapy for a parotid tumour and outlines a systematic approach for fetal dose estimation, optimization and verification.

## Case presentation

A 27-year-old female patient presented in October 2023 with left-sided hemifacial pain, left facial nerve palsy, and a general decline in health, including an unintentional weight loss of 10  kg over the course of three months. At that time, the patient reported a confirmed pregnancy since August 5, 2023.

Cervical computed tomography (CT) images and magnetic resonance (MR) images showed a 31 × 23 mm intra-parotid mass on the left side, encasing the facial nerve, without nodal or distant disease. Core needle biopsy confirmed classic-type adenoid cystic carcinoma.

Following local multidisciplinary tumor board discussion, upfront surgical management was recommended. The patient underwent a left total parotidectomy with selective homolateral neck dissection encompassing levels Ia, Ib, IIa, III, and IV. Final histopathology revealed a 4  cm high-grade tumor with a proliferative index of 80% (Ki-67) and marked perineural invasion. Two metastatic lymph nodes were identified: one *peri*-parotid node and one level IIb node, both without extracapsular spread. The pathological staging was pT4a N3b (2/32 lymph nodes). Surgical margins were classified as R1, with tumor involvement at the facial nerve pathway.

Given the pregnancy, the case was presented at a national multidisciplinary tumor board organized by the French Rare Head and Neck Cancer Network (REFCOR). After also considering the ICRP recommendations (2) and previously reported cases in the literature [3], [5], adjuvant radiotherapy without chemotherapy was recommended. Following ethics committee consultation and prenatal counseling regarding fetal risks, the patient opted to continue the pregnancy.

Radiotherapy treatment began in January 2024, (26–––32 weeks’ gestation). The prescribed and delivered doses were 52.8  Gy to the ipsilateral prophylactic lymph node areas and 66  Gy to the high-risk areas (surgical bed and involved lymph nodes) using a simultaneous integrated boost over 33 fractions. To our knowledge, this was the first such case at our institution. An extended preparation time was agreed upon by the medical and medical physics teams (1  month instead of the usual 15  days between simulation and treatment start) to ensure optimal planning without compromising tumor control.

Simulation imaging was performed conventionally, with the scan range limited inferiorly to the tracheal carina. No lead shielding was used at this stage. No fetal dose estimation was performed for the CT simulation. The position of the uterine fundus was assessed via ultrasound and marked on the skin. The distance between this skin mark and the patient’s clavicle was then measured during the simulation. Finally, the cranio-caudal distance from the isocenter to the clavicle was manually measured on the CT images, allowing the distance from the isocenter to the uterine fundus to be deduced.

### Dosimetric Optimization

Although out-of-field doses are generally lower with conformal radiotherapy than with modulated radiotherapy [1], volumetric modulated arc therapy (VMAT) was chosen to maintain optimal plan quality, ensuring superior target conformity, dose homogeneity, and sparing of OARs.

Shortly before our patient’s treatment, a study on radiotherapy planning in the context of pregnancy was published by the 10.13039/501100020711French Institute for Radiological Protection and Nuclear Safety (10.13039/501100004598IRSN, now 10.13039/100011975ASNR) [13]. The study showed that the collimator angle of the linear accelerator could induce significant variation in the out-of-field dose and can be a parameter of interest for fetal dose reduction. This work also demonstrated that by optimizing VMAT plans, it is possible to reduce fetal exposure to doses similar to those obtained with conformal radiotherapy for a cranial tumor.

Based on these findings, three clinically equivalent VMAT plans (P_ref_, P_opt1_, P_opt2_) were developed using two full coplanar arcs with contralateral avoidance sectors and 6 MV photons on a TrueBeam linear accelerator (Varian Medical Systems, Palo Alto, CA, USA) equipped with a 120-leaf Millennium collimator. The optimized treatment plan was equivalent to a classical one in terms of target coverage and OAR Dose Volume Histograms. Therefore, no additional toxicity was expected. Minimal variations in monitor units (MU) were observed (P_ref _= 449 MU, P_opt1 _= 460 MU, P_opt2 _= 487 MU). The main difference was the collimator angle: 15°/345° (P_ref_), 80°/30° (P_opt1_), and 80°/100° (P_opt2_).

**Fig. 1:**
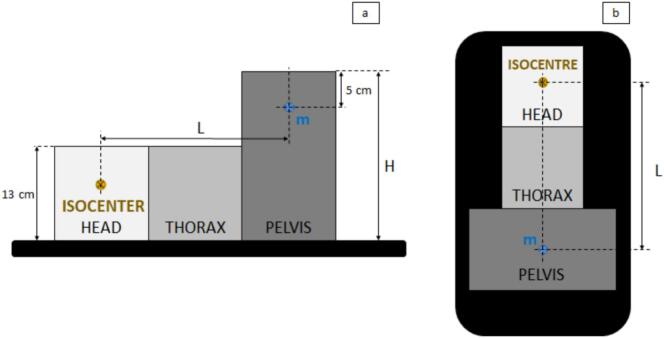
Diagram of the experimental setup with the three phantom slab blocks (Head, Thorax and Pelvis), shown in sagittal view (a) and coronal view (b), relative to a standard supine treatment position on a linear accelerator. The position of the measurement point m varied according to the values of distances L and H.

Fetal dose estimation for the three plans used a water-equivalent slab phantom and a PTW Farmer 0.6  cm^3^ ionization chamber (Fig. 1). The phantom included head–thorax (30 × 30  cm) and pelvic (30 × 40  cm) blocks. The cranio-caudal distance L between the isocenter and uterine fundus was 45  cm, with the measurement point m set 5  cm deep in the pelvic block, centered laterally.

**Fig. 2:**
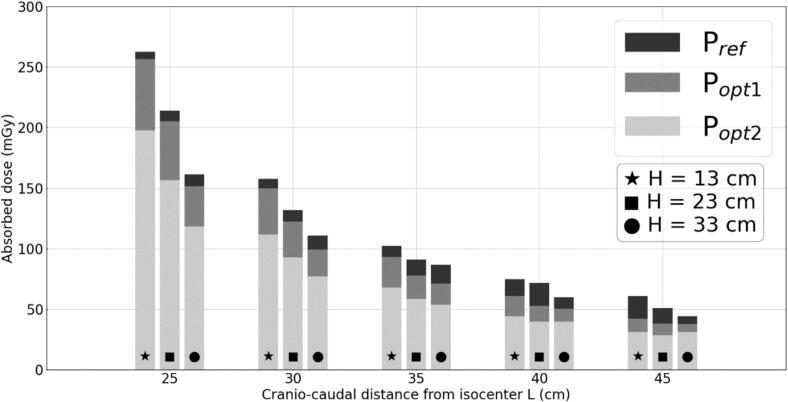
Histogram of the out-of-field dose variation measured at point m and extrapolated to the entire treatment, as a function of the distance from isocenter (L) and the height (H) of the pelvis block, for the three plans: P_ref_, P_opt1_ et P_opt2_.

In order to simulate possible changes related to the pregnancy progression, such as an increase in uterine height or abdominal thickness, measurements were performed at different cranio-caudal positions (L from 45 to 25  cm, in 5  cm steps) and varying anterior-posterior thicknesses (pelvic block height H from 13 to 33  cm, in 10  cm steps). These configurations enabled the creation of a reference chart to estimate fetal dose in case of significant anatomical changes during treatment. Each measurement corresponded to a single treatment fraction, but results (presented in Fig. 2) were extrapolated to the full course of 33 fractions.

For all plans and configurations, a consistent increase in dose was observed as the distance L decreased, with a maximum fivefold increase when going from 45 to 25  cm. Similarly, a reduction in block height H resulted in a consistent increase in dose, of around 70% from 33  cm to 13  cm. The combination of these two factors further amplified the dose increase. Regardless of the scenario, measurements for P_opt2_ were consistently lower than those for the other plans. Except for the configuration with L = 25  cm, P_opt2_ remained below or close to the 100  mGy threshold. After careful consideration of these results, P_opt2_ was selected for treatment.

During optimization with P_opt2_, adding a 0.5  mm lead-equivalent lead apron over the phantom’s pelvis (Burlington Medical Supplies) reduced dose by 5–13%. Consequently, with the agreement of the radiation oncologist and the patient, the apron was used to cover the abdomen throughout treatment.

### Treatment and *in vivo* dosimetry

During treatment, daily repositioning was achieved using two orthogonal kV X-ray images to minimize imaging dose compared with CBCT. To closely monitor possible soft tissue variations which are more difficult to visualize with this imaging modality, increased monitoring of the patient’s weight and mask fit was implemented.

*In vivo* fetal dose monitoring was performed during the last two treatment weeks using radiophotoluminescent (RPL) dosimeters (GD-301, Technol). The detectors were placed beneath the lead apron, positioned at the abdominal surface corresponding to the uterine fundus. Weekly ultrasound imaging was used to verify and, if necessary, adjusted this reference mark, while abdominal thickness was measured to compare with phantom-based estimations. Both parameters varied by less than ± 2 cm throughout the seven-week course.

Two sets of three dosimeters were used: one for single-session measurements (10 sessions) and another for cumulative weekly doses (five sessions per week). Mean cumulative doses were 5.9  mGy and 6.7  mGy for the last two weeks, while single-session measurements averaged 1.3  mGy. Extrapolation over the full treatment yielded an estimated fetal dose of ∼ 41.0  mGy, consistent with a phantom-based calculation of 31.1  mGy after adjustment for patient-specific anatomical parameters.

## Discussion and Conclusions

Radiotherapy was completed without adverse events. The patient received clinical medical monitoring at least weekly throughout the entire duration of the treatment. The acute toxicities were limited to grade I xerostomia, grade I dysgeusia and grade II dermatitis. Follow-up consultations at 6  weeks, 3  months post-treatment and then every three months showed the resolution of these acute toxicities with complete recovery of skin and salivary gland reactions. Only the post-surgical facial paralysis persisted, as expected. Nutritional monitoring was conducted weekly by the dietetics department of our center.

The patient tolerated both dosimeter placement and the lead apron well. During the implementation of the *in vivo* dosimetry, the patient was systematically asked whether she felt any pain or inconvenience from having the lead apron and dosimeter on her abdomen. Each time, the patient did not report any discomfort and even expressed reassurance regarding the implementation of *in vivo* dosimetry and lead apron usage.

Monthly follow-up with fetal ultrasound was performed throughout the entire pregnancy, including during the course of radiotherapy, by her attending obstetrician. This follow-up remained entirely normal. A healthy baby was born in April 2024, with normal neurological and developmental outcomes at 15  months. A standard pediatric follow-up was recommended, with particular attention paid to the child’s proper cognitive development.

This unprecedented case established a foundation for our department’s methodology to estimate and minimize fetal dose. This strategy is part of a dose-reporting framework for long-term child follow-up, combining phantom-based experiments and *in vivo* dosimetry. A sufficiently low fetal dose level, consistent with international guidelines (< 100  mGy), was both estimated and validated for the treatment. While all three tested treatment plans remained under this threshold, the plan with the lowest estimated fetal dose (P_opt2_) was selected as part of the optimization process. The optimization confirmed the previously described influence of collimator angle selection in VMAT technique, as outlined by Edouard et al. [13]. Compared to the reference plan (P_ref_), no compromises in dose conformity or homogeneity to the target volumes, nor in OARs sparing, were required.

Phantom and *in vivo* RPL dosimetry yielded comparable fetal dose estimates, 31  mGy and 41  mGy, respectively, for the full treatment. Direct comparison was limited by differences in detector depth (surface vs. 5  cm), anatomical geometry, and detector energy response. Despite these factors, phantom measurements proved valuable for plan comparison and preliminary dose estimation, while their consistency with *in vivo* results supports the reliability of the overall assessment.

Many clinical cases involving radiotherapy in pregnant women include fetal dose estimation. Most teams use anthropomorphic phantoms and/or water-equivalent slabs for fetal dose measurement. The reported dose levels are similar to our findings, generally ranging from 10 to 90  mGy depending on treatment site, equipment, and technique [8].

Focusing specifically on head and neck treatments, Marchesi et al. estimated a fetal dose below 20  mGy for parotid irradiation performed with conformal non-modulated radiotherapy [3]. Moeckli et al. estimated a fetal dose of 36  mGy also using conformal radiotherapy, including contributions from diagnostic imaging [5].

Reports on *in vivo* dosimetry in pregnant women remain rare. In addition to phantom measurements, Antypas and Moeckli’s teams used thermoluminescent detectors (TLDs) placed in the vaginal canal and rectum, respectively, to perform *in vivo* measurements during conformal treatments to the breast [14] and head and neck region [5]. Antypas’s group further refined fetal dose estimation by using an ionization chamber placed in the rectum. In both studies, a level of agreement comparable to ours was observed between phantom-based and *in vivo* measurements. Specifically, Antypas reported estimated fetal doses of 3.9  mGy, 3.6  mGy, and 3.8  mGy respectively for *in vivo* with TLDs, *in vivo* with the ionization chamber, and phantom-based with the ionization chamber. Moeckli’s study reported doses of 10.8  mGy *in vivo* (TLDs) and 10.9  mGy on phantom (ionization chamber).

This study had several limitations. The phantom setup simulated abdominal thicknesses up to 33  cm, constrained by the available water-equivalent slabs. Lateral displacement relative to the isocenter (≈ 4  cm toward the treated side) was not assessed, and the use of a more patient-representative anthropomorphic phantom could further improve accuracy.

To better validate *in vivo* dosimetry, it would have been valuable to investigate the correlation between surface-level RPL measurements and those taken in depth with the ionization chamber in the phantom.

Finally, fetal dose from diagnostic imaging was also not assessed, though previous work suggests it may exceed therapeutic contributions [5].

In conclusion, this clinical case represents our team’s first experience managing a pregnant patient undergoing radiotherapy. We successfully implemented a treatment beam configuration optimization using a phantom to limit fetal exposure—consistent with international guidelines—and performed *in vivo* dosimetry to validate phantom-based estimates. This approach enabled us to report and document the estimated fetal dose for comprehensive clinical and ethical follow-up. The experience established a practical and ethical framework for managing similar cases and supports the feasibility of using modern, highly conformal radiotherapy techniques during pregnancy when clinically indicated.

## Author CRediT statements

All the authors read and approved the final manuscript. AM: Conception and design of the work. Acquisition, analysis and interpretation of the data work. DT: Patient management and follow-up. LD: Dosimetric planning and optimization. LA: Analysis and interpretation of the data work. KP: Analysis and interpretation of the data work. CH: Provision, analysis and interpretation of RPL measurements. ME: Provision, analysis and interpretation of RPL measurements. JB: Revision of the work for important intellectual content. Analysis and interpretation of the data work. IB: Conception and design of the work. Acquisition, analysis and interpretation of the data work.

## Patient consent Statement

The patient and his family approved the collection of data for publication purpose by signing a consent form.

## Declaration of competing interest

The authors declare that they have no known competing financial interests or personal relationships that could have appeared to influence the work reported in this paper.
